# Quantitative evidence of suppressed TMEM119 microglial immunohistochemistry in fatal morphine intoxications

**DOI:** 10.1007/s00414-021-02699-5

**Published:** 2021-09-22

**Authors:** Simone Bohnert, Kosmas Georgiades, Camelia-Maria Monoranu, Michael Bohnert, Andreas Büttner, Benjamin Ondruschka

**Affiliations:** 1 Institute of Forensic Medicine, University of Wuerzburg, Versbacher Str. 3, 97078 Wuerzburg, Germany; 2 Department of Neuropathology, Institute of Pathology, University of Wuerzburg, Josef-Schneider Str. 2, 97080 Wuerzburg, Germany; 3 Institute of Legal Medicine, Rostock University Medical Center, St.-Georg-Strasse 108, 18055 Rostock, Germany; 4 Institute of Legal Medicine, University Medical Center Hamburg-Eppendorf, Butenfeld 34, 22529 Hamburg, Germany

**Keywords:** Drug abuse, Forensic neuropathology, Neuroinflammation, Neurotoxicity, Microglia, Immunohistochemistry

## Abstract

The aim of this pilot study was to investigate the diagnostic potential of TMEM119 as a useful microglia-specific marker in combination with immunostainings for phagocytic function and infiltrating capacity of monocytes in cases of lethal monosubstance intoxications by morphine (MOR), methamphetamine (METH), and of ethanol-associated death (ETH) respectively. Human brain tissue samples were obtained from forensic autopsies of cases with single substance abuse (MOR, *n* = 8; ETH, *n* = 10; METH, *n* = 9) and then compared to a cohort of cardiovascular fatalities as controls (*n* = 9). Brain tissue samples of cortex, white matter, and hippocampus were collected and stained immunohistochemically with antibodies against TMEM119, CD68KiM1P, and CCR2. We could document the lowest density of TMEM119-positive cells in MOR deaths with highly significant differences to the control densities in all three regions investigated. In ETH and METH deaths, the expression of TMEM119 was comparable to cell densities in controls. The results indicate that the immunoreaction in brain tissue is different in these groups depending on the drug type used for abuse.

**Supplementary Information:**

The online version contains supplementary material available at 10.1007/s00414-021-02699-5.

## Introduction

Investigations of drug deaths including neuropathological diagnostics are important tasks of daily forensic work. The methodological problem in evaluating the effects of drugs of abuse on the central nervous system (CNS) consists of distinguishing between substance-specific effects related to the properties of the drug itself, the influence of neurotoxic adulterants/diluents, and of secondary effects related to the lifestyle of drug abusers, for example, malnutrition, infections, and systemic diseases [1, 2]. Since the majority of drug abusers perform polydrug abuse, including ethanol and nicotine, it is nearly impossible to link distinct neuropathological findings to single substances [3, 4].

Microglia have long been recognized as “sensors of pathology.” As the major cells of the brain’s immune system, one of the primary functions of microglia is to be the first line of defense and to engage in neuroprotection whenever necessary [5–9]. When the neuronal activity is disturbed or compromised by injury, disease, or neurotoxic agents, they become activated by changing into an amoeboid morphology, primarily acting in a phagocytic/macrophage fashion and being difficult to differentiate from infiltrating peripheral macrophages [10, 11].

The most widely used microglia markers (ionized calcium–binding adaptor molecule 1-Iba1 and CD68) in brain tissue slides do not allow any differentiation between resident microglia and infiltrating blood-derived macrophages [12]. The robustly expressed trans-membranous molecule TMEM119 is known as a microglia-specific marker, which is not expressed by other macrophages, immune or neuronal cells, and is, therefore, the most promising microglia marker to date [12, 13]. TMEM119 has served as a reliable immunohistochemical microglia marker in forensic assessments of traumatic causes of death, e.g., TBI [14], and was even positively stained by an adapted protocol for immunocytochemistry in postmortem CSF samples of trauma cases [15]. However, its function and involvement in the brain’s injury response are yet to be defined [16].

In general, the involvement of glial cells as important players in drug-induced synaptic plasticity and neuroinflammation has long been neglected [3]. Drug abuse could influence glia directly through interactions at surface receptors sensitive to these natural or synthetic compounds, or indirectly through their effects on neighboring neurons surveyed by microglia or astrocytes [17].

Derivatives of opium, particularly morphine (MOR), have been used for thousands of years due to their potent analgesic and rewarding effects. Apart from their effects on neurons, there is some evidence that microglia can be influenced through classical opioid receptor (*μ*, *δ*, *κ*) signaling. Cell cultures of neonatal cortical microglia from rats show immunoreactivity for all three receptor classes and dynamically regulate their expression in response to morphine exposure [18]. Ethanol (ETH) may induce neuroinflammation. Increases in microglial markers, i.e., IBA1, have been observed in mouse brain following binge or chronic ethanol drinking and in human postmortem brain from ethanol depended fatal cases [19]. Chronic abuse of methamphetamine (METH) is a feature with neurotoxicity marked by diminished dopamine concentration, low level of dopamine transporter, and neuroinflammation. There has been much speculation about how METH toxicity may directly affect microglia activation and if microglia activation contributes to brain damage [20]. However, most of our knowledge on the neurobiological basis and consequences of drug abuse has been derived from animal models [3].

In the past, there were only few detailed postmortem studies of neuroinflammation in human drug abusers, predominantly based on polydrug abuse and focused on the consequences of hypoxia–ischemia or cerebrovascular events; therefore, little is known about the fundamental neuropathological alterations of the cellular elements [3]. The aim of this pilot study was to investigate for the first time the potential of TMEM119 as a useful microglia-specific marker in a “forensic preselection” of *fatalities with single substance abuse* (MOR, ETH, METH) and to evaluate microglial contribution to brain pathology in respect to its cellular diversity in combination with the phagocytic function (stained by CD68KiM1P immunohistochemistry) and the infiltrating capacity of monocytes (using CCR2 antibodies).

## Material and methods

### Sampling and processing

Human brain tissue samples were obtained from autopsies performed at the Institute of Forensic Medicine, University of Wuerzburg, and the Institute of Legal Medicine, University of Leipzig. This research study has been approved by the ethics committee of the Medical Faculty of the University of Wuerzburg (local number 203/15) and by the ethics committee of the Medical Faculty of the University of Leipzig (local numbers 117–12-23,012,012 and 328–08). Thirty-six cases were included in this study, 8 females and 28 males; the age at death ranged from 22 to 85 years. The samples were divided into cases with analytical-proved lethal intoxications with monosubstances (MOR, METH), and death cases happened under the influence of ethanol (ETH) (total number *n* = 27; *n* = 8 MOR, *n* = 10 ETH, *n* = 9 METH; case characteristics are displayed in detail in Table 1), and compared to a cohort of cardiovascular fatalities as controls (total number *n* = 9) with their postmortem interval (PMI) varying between 1 and 6 days. The history of drug respectively ethanol abuse, CNS trauma, and circumstances of death have been derived from the clinical (as far as available) and the police records. In all cases, toxicological analyses were performed on blood, urine, and gastric content. In addition, blood ethanol concentrations were measured in every case. Ethanol determination was performed in each case as a duplicate determination and averaging by headspace gas chromatography and enzymatic conversion with alcohol dehydrogenase. Morphine determination was performed after solid-phase extraction and derivatization with pentafluoropropionic anhydride and pentafluoropropanol by gas chromatography–mass spectrometry. Methamphetamine determination was performed after liquid/liquid extraction and derivatization with pentadecafluoroctanoyl chloride by gas chromatography–mass spectrometry. All control cases were tested negative for illicit drugs and ethanol.

**Table 1:** Characteristics of single substance (MOR, morphine; ETH, ethanol; METH, methamphetamine) and control cases investigated in this study

Case number	Sex	Age	PMI	Cause of death	Concentration
				Mono intoxication	in ng/ml (blood)
1	m	27	4	METH	836
2	m	28	5	METH	3100
3	m	28	4	METH	558
4	m	33	4	METH	1336
5	m	33	3	METH	17,400
6	m	36	5	METH	1296
7	m	43	3	METH	293
8	m	44	2	METH	3957
9	m	46	4	METH	2718
1	m	39	5	MOR	670
2	m	24	5	MOR	111
3	m	43	6	MOR	144
4	m	73	6	MOR	366
5	m	35	5	MOR	121
6	m	31	5	MOR	180
7	f	55	6	MOR	427
8	m	26	4	MOR	200
				Ethanol-associated death	in ‰ (blood)
1	m	29	4	ETH	1.34
2	f	42	2	ETH	1.68
3	m	35	3	ETH	1.43
4	m	50	4	ETH	0.71
5	m	67	3	ETH	2.61
6	m	64	2	ETH	0.72
7	m	49	1	ETH	2.14
8	m	71	1	ETH	1.35
9	f	60	1	ETH	2.61
10	m	77	2	ETH	0.77
				Cardiovascular fatalities	Underlying disease
1	f	51	1	Acute myocardial infarction	Coronary artery disease
2	f	71	2	Acute myocardial infarction	Coronary artery disease
3	m	85	1	Acute myocardial infarction	Coronary artery disease
4	m	66	1	Acute myocardial infarction	Coronary artery disease
5	m	35	3	Ruptured aortic aneurysm	Suspected cystic mediadegeneration
6	m	22	1	Ruptured aortic aneurysm	Suspected Marfan syndrome
7	f	78	1	Acute myocardial infarction	Coronary artery disease
8	f	38	1	Myocarditis	Myocarditis
9	f	46	2	Acute myocardial infarction	Coronary artery disease

Brain specimens of cortex, white matter and hippocampus were collected retrospectively and fixed in neutral buffered 4% formalin, then embedded in paraffin. The period of fixation varied between a few days and 4 years. Cerebral samples included cortical layers and white matter. Hippocampus samples were chosen as a CNS region being vulnerable to ischemic-hypoxic episodes [21, 22] as well as a region known for high density of microglia. After paraffinization, the wax blocks were sliced at 6 µm using a microtome. Consecutive sections were mounted on microscope slides and stained immunohistochemically, as previously described [23], with commercially available antibodies against TMEM119 in a dilution of 1:1000 (Sigma, St. Louis, USA), against CD 68Kim1P in a dilution of 1:200 (Abcam, Berlin, Germany) and CCR2 in a dilution of 1:200 (Abcam, Berlin, Germany) as primary antibodies. The MultiLink Streptavidin-Peroxidase-Kit (BioGenex, San Ramon, USA) was used as secondary antibody. Control slides were stained by omitting the primary antibodies given above to test for unspecific staining in all staining charges. The microphotographs of the brain sections were taken with a Leica digital camera DMC 5400 mounted on a Leica DM6 B microscope using 100 × magnification constantly (both Leica Microsystems Corporation, Wetzlar, Germany).

For the slides with different antibodies investigated, five randomized images each per slide were taken to obtain a representative surface for all sections. The total surface of the images equals 5.8 mm^2^ (1.16 mm^2^ per single photograph). For quantitative evaluation of the sections, an image processing software (Leica LASX, Wetzlar, Germany) was used as described before [14, 24]. Prior to the electronic count, parameters of cell morphology (size and staining intensity) were defined for each antibody, which were not changed throughout the evaluation. The software transferred the data into an Excel-macro table (Microsoft Corporation, Redmond, USA) automatically. The number of cells discernible in the five fields of view (profile density) was set against the area investigated and calculated as the number of immunopositive cell perikarya per square millimeter or density per mm^2^.

### Statistical analysis

Excel Version 16.15 (Microsoft Corporation) and GraphPad Prism software version 8 (GraphPad Software, La Jolla, USA) were used for statistical evaluation. The Shapiro–Wilk normality test was used to test the distribution of the samples. Parametric data of samples were then tested using an ordinary one-way ANOVA with post hoc Tukey’s multiple comparisons test subsequently. A Kruskal–Wallis test was used for nonparametric data followed by Dunn’s test to avoid repetitive testing failure. Adjusted *p* values equal to or smaller than 0.05 were considered statistically significant.

## Results

**Fig. 1:**
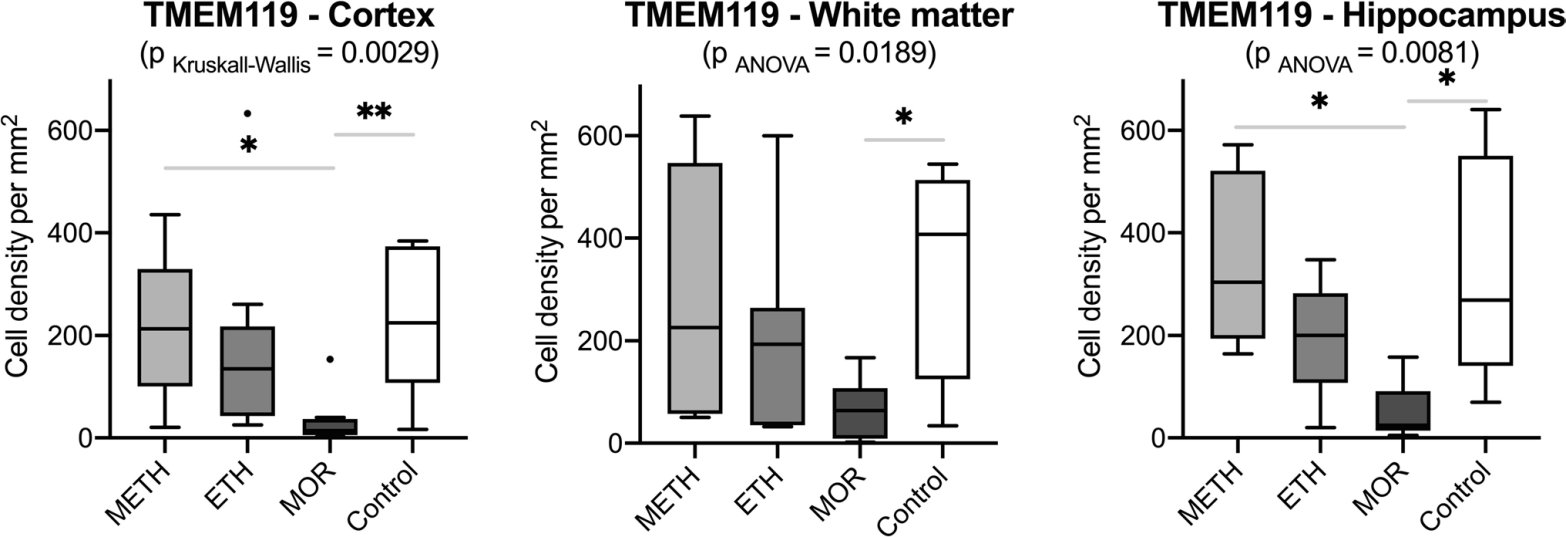
Box plot diagrams displaying the different total densities of TMEM119-positive microglial cells (counted in five digital images at a 100 × magnification) in METH, ETH, and MOR cases compared to the controls in the three brain regions investigated. The solid black lines indicate the median, the outlines of the boxes the 25^th^ and 75^th^ percentile. Whiskers are defined as Tukey’s end of 1.5 times interquartile range and all outliers are illustrated as dots outside these fences. ^*^p < 0.05; ^**^p < 0.001

**Fig. 2:**
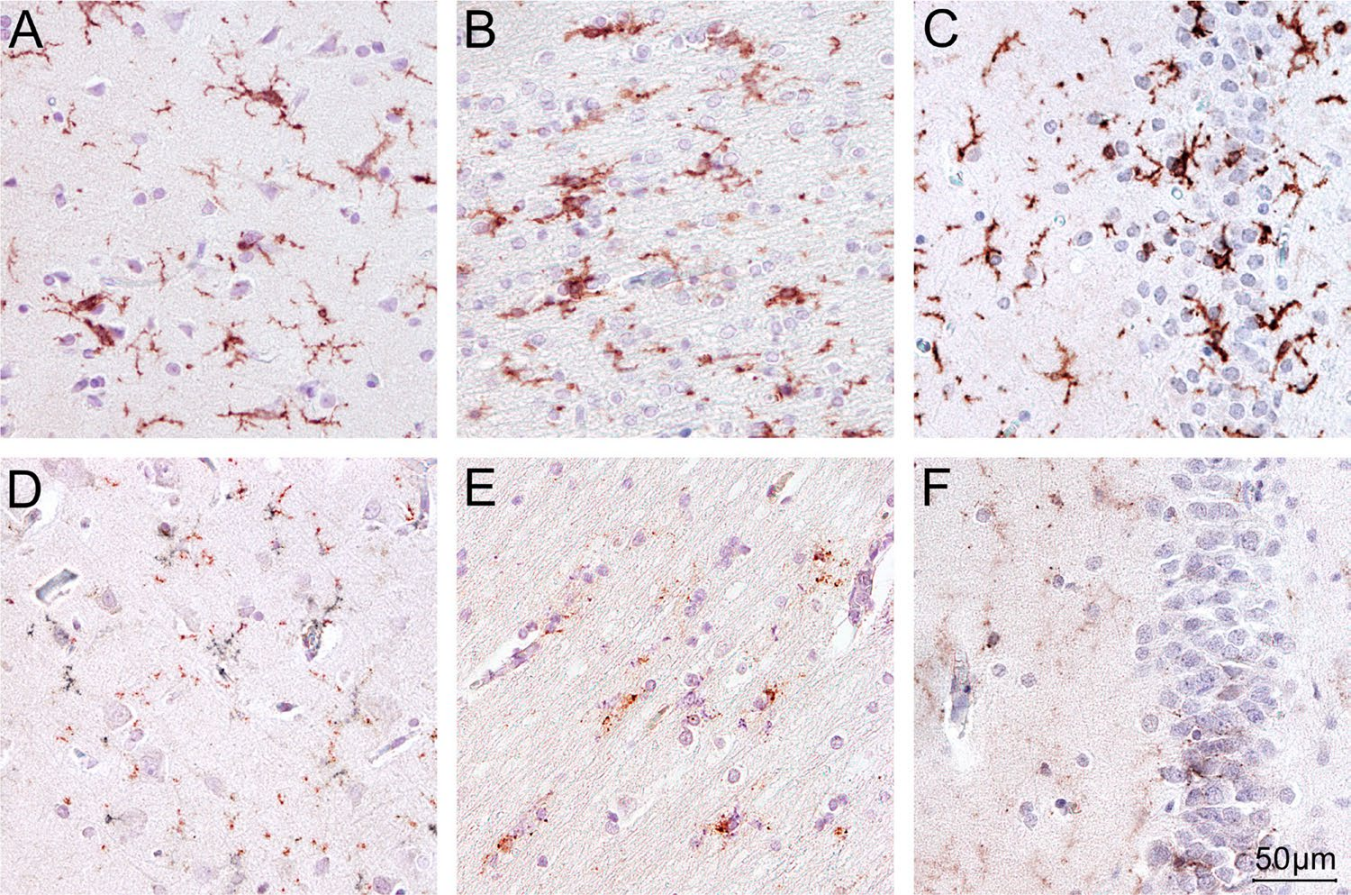
Examples of immunohistochemical staining (IHC) results using TMEM119 in the cortex (**A**), the white matter (**B**), and the hippocampus (**C**) in control cases and in the cortex (**D**), the white matter (**E**), and the hippocampus (**F**) in MOR cases. In the control cases both, microglial perikarya and processes are visible. The perikarya appear more or less completely filled with partly coarse- and partly fine-granuluar TMEM-positive material. IHC-positive puncta are mainly encountered in the glial processes with preference for branching points of processes. In the MOR cases, low density fine-granular IHC-positive profiles predominate, and it is difficult to decide whether these profiles are located in the microglial perikarya or in the processes of the cells. Magnification: 200 ×

**Fig. 3:**
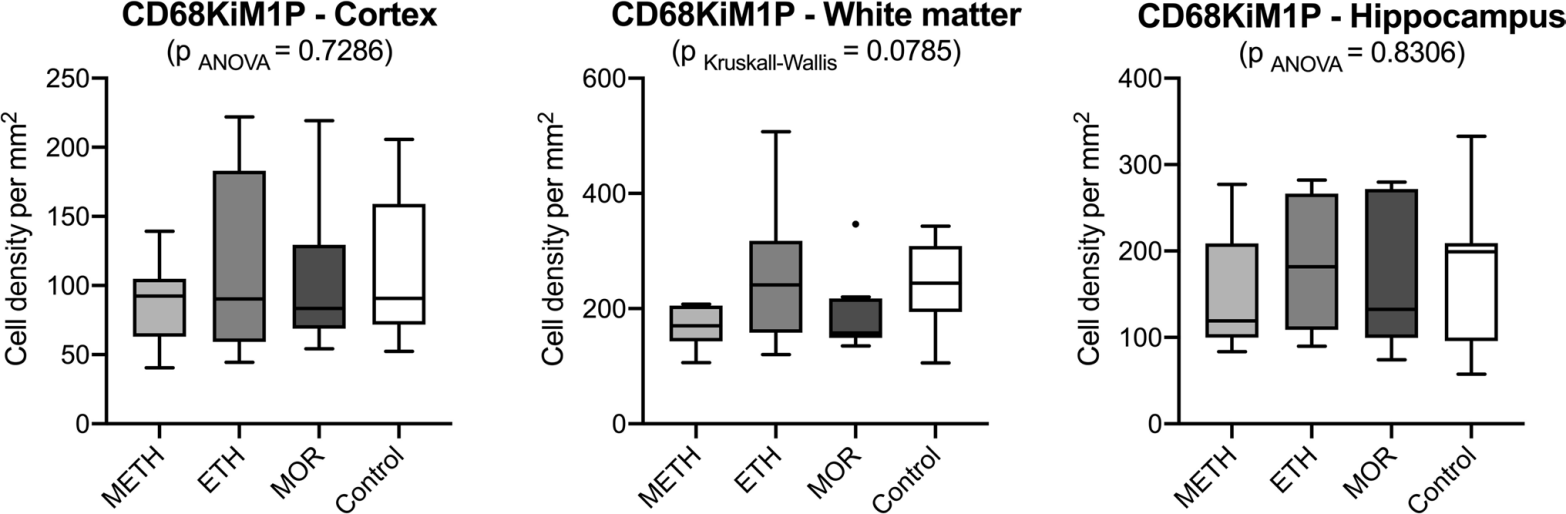
Box plot diagrams displaying the different total densities of CD68KiM1P-positive cells (counted in five digital images at a 100 × magnification) in METH, ETH, and MOR compared to the controls in the three brain regions investigated. The solid black lines indicate the median, the outlines of the boxes the 25^th^ and 75^th^ percentile are illustrated as dots outside these fences. No statistically significant results were obtained

**Fig. 4:**
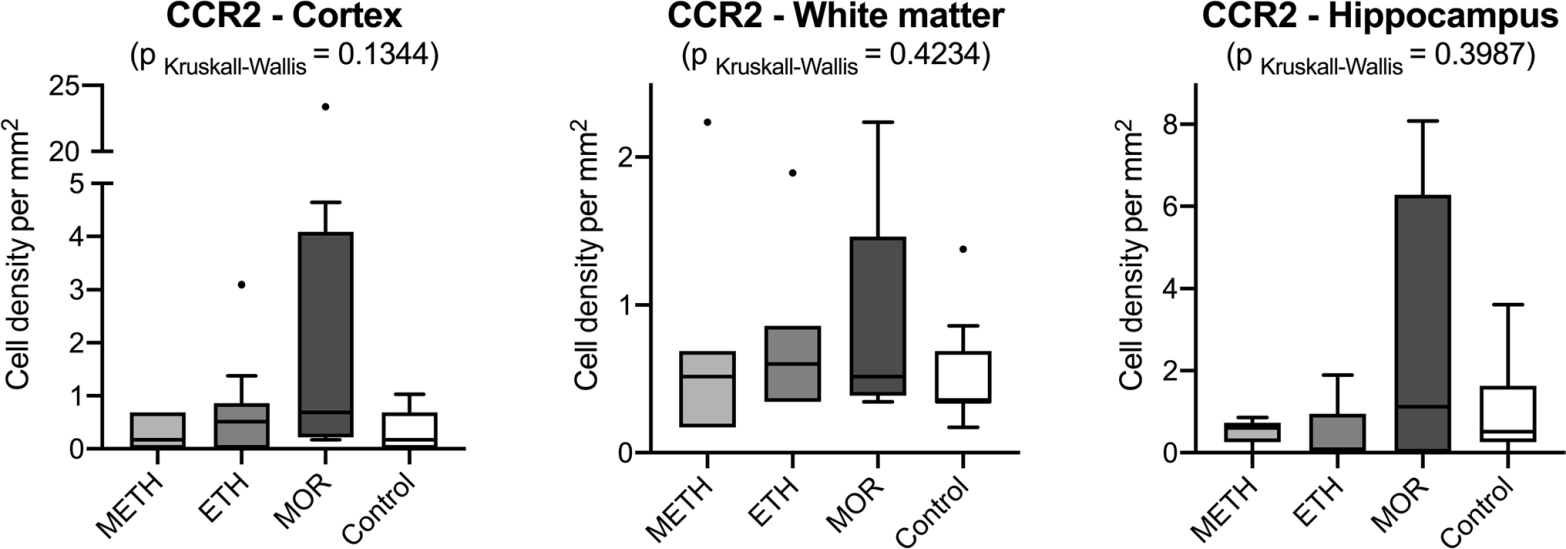
Box plot diagrams displaying the different total densities of CCR2-positive cells (counted in five digital images at a 100 × magnification) in METH, ETH, and MOR compared to the controls in the three brain regions investigated. The solid black lines indicate the median, the outlines of the boxes the 25^th^ and 75^th^ percentile. Whiskers are defined as Tukey’s end of 1.5 times interquartile range and all outliers are illustrated as dots outside these fences. No statistically significant results were obtained

When comparing all cases investigated (MOR, ETH, METH, and controls), the lowest density of TMEM119-positive cells were counted in MOR cases (all with significant differences to the control densities) in all three investigated brain regions. ETH and METH cases did not show different immunostaining counts when compared to each other or to the control samples. Particularly in the cortex, there was a significant reduction of microglia in MOR cases as compared to the control group and the other investigated substances (see Fig. 1). Typical examples of immunohistochemical staining results (IHC) of TMEM marker used in MOR (Fig. 2D–F) and controls in different regions investigated (Fig. 2A–C) are presented in Fig. 2. Both the density of IHC-positive microglial processes and that of the microglial perikarya (cytoplasm surrounding microglial nuclei) were reduced qualitatively. CD68 KiM1P–positive macrophages were nearly equally distributed within all brain regions examined and did not show any significant differences of the positive cell count compared to the other investigated substances or the control group (Fig. 3). Typical IHC staining results of CD68KiM1P marker used in controls (Fig. 1A–C) and MOR cases (Fig. 1D–F) in different regions investigated are presented in Fig. 1, see Supplemental. Both CD68KiM1P IHC-positive microglial perikarya and processes could be distinguished in the cerebral cortex of control (Fig. 1A) and in the MOR cases (Fig. 1D). In sections of the cortical central medullary ray (Fig. 1B/E) and the dentate fascia (Fig. 1C/F), the density of microglial profiles displaying the characteristic arborizing processes decreased, whereas the density of dot-like profiles and microglial perikarya without terminal ramifying processes increased. CCR2-positive profiles with monocytic phenotype were rarely encountered in ETH and METH cases as well as in controls. In MOR cases, the density of CCR2-positive profiles was enhanced. However, the individual variation of the number of profiles per counting field resulted in statistically non-significant differences between cases as well as controls (Fig. 4). Typical IHC staining results of CCR2 marker used in controls (Fig. 2A–C) and MOR cases (Fig. 2D–F) in different regions investigated are presented in Fig. 2, see Supplemental.

## Discussion

In our study, we focused on the resident microglia because their immediate response to traumatic or non-traumatic tissue damage plays a central role in CNS neuroinflammation [5, 8, 9]. Similarly, previous studies have shown that resident microglia have a different distribution pattern with the highest density of cells in basal nuclei, substantia nigra, and hippocampus [17, 22]. In drug-associated deaths, microglial cells have been described in white matter and subcortical areas [3]. However, to date, little is known about microglial changes following chronic and/or acute substance abuse. A mechanistic analysis and understanding of drug actions on human central nervous system is bedeviled by brain asymmetry, gender differences, individual variability, and selective vulnerability. Data collected from animal models apply to humans only to a limited extent [25].

Opioids are among the most frequently consumed substances. Abuse of opioids is known to cause death by suppression of respiratory brain stem centers [3]. Traditionally, MOR was defined as an immunosuppressive drug. Chronic morphine-dependent subjects are afflicted by various infectious diseases [26]. Although many basic functions of the innate and the adaptive immune system such as phagocytic activities of macrophages or T-cell, B-cell antibody response were inhibited by morphine in the animal model, the production of proinflammatory cytokines is, in general, increased by morphine administration [27]. The immune modulation of morphine is more complicated. An alternative explanation for morphine-induced vulnerability to infection is the impairment of pathogen clearance capability while augmenting pathogenic inflammatory response. Wang et al. (2012) demonstrated in a mouse model that morphine administration systematically dampens the activation of the peripheral immune system, However, the CNS-resident immune cells or microglia were overactivated [28]. Various studies have suggested that such effects of MOR may either be via classical opioid receptors or via an interaction somewhere along the intracellular cascade activated by Toll-like receptor 4 (TLR4) [28].

In the present pilot study, we used the microglia-specific antibody TMEM119 to investigate the neuroinflammatory response of brain tissue after fatal monosubstance abuse compared with cardiovascular controls in order to differentiate between resident microglia and infiltrating blood-derived macrophages.

The ingestion of ETH and METH monosubstances was not associated with a decreased density of TMEM119-positive perikaryal profiles compared to the control group. On the other hand, the density of TMEM119-positive perikaryal profiles was significantly reduced in the cortex, white matter, and hippocampus of cases with MOR intoxication. A similar effect was observed in the white matter of multiple sclerosis (MS) cases. In MS, this phenomenon was explained by cytokines in local inflammatory environment and confirmed by cell culture observations [29]. In addition, TMEM119 mRNA levels were reduced in mouse-derived microglia treated with lipopolysaccharide in vitro indicating that the expression of the marker can be regulated by inflammatory cytokines [13]. It remains to be investigated whether similar factors are responsible for the selectively reduced cell density of TMEM-positive microglial cells in MOR cases and why TMEM119 microglial density is not reduced in the given METH and ETH cases.

The quantitative deficit of TMEM119-positive perikarya could not be observed after staining with the “pan marker” CD68KiM1P. No differences were seen between the individual monosubstances and the control group. The “pan marker” CD68KiM1P positively marks both macrophages as well as resident microglia according to the manufacturer’s handsheet. Consequently, the density of immunopositive profiles should be higher than those of exclusively TMEM119-immunostained sections in theory. The interpretation of our data is hampered by the great variation including outliers in our cases. Only the estimation of TMEM119-positive cells in cortex, white matter, and hippocampus of our MOR cases compared with the density of CD68-positive profiles in identical regions proofed to be lower. Given the CD68 reaction is a genuine microglial pan marker, it remains to be verified in the future that our quantitative results represent a disease-specific microglial cocktail reacting to different toxic substances.

In addition to overall reduced TMEM-positive perikarya density, subtle qualitative differences in the outcome of our immunohistochemical protocols were observed. Immunopositive microglial cell profiles with chromophilic perikaryon and delicate ramifying peripheral processes could be diagnosed after TMEM119 and CD68KiM1P immunohistochemical staining in the cortex and white matter of control cases. The most delicate peripheral processes were best displayed after TMEM-119 immunohistochemistry in control cases, whereas local- and disease-specific differences in the perikaryon could be recognized after CD68KiM1P staining. CD68-positive profiles could be unequivocally detected in cortex and white matter of our control cases. However, in the MOR subjects included, TMEM119-positive microglial perikarya were difficult to delineate in all three regions investigated given that finely granular TMEM-119-positive profiles were difficult to attribute to either perikarya or predominantly peripheral microglial terminal processes, however.

In general, immunohistochemistry is subjected to artefactual changes including shrinkage and swelling of tissue sections. Swelling can result in reduced density of immunopositive profiles, whereas shrinkage may increase the density of immunopositive results. These artefacts are difficult to control and they must be always kept in mind when interpreting data, especially when using postmortem tissue [30].

To differentiate between resident microglia- and blood-derived macrophages, we studied the cell densities of CCR2-positive monocytes in our cases investigated. We could document the highest cell densities of CCR2-positive monocytes in the MOR cases compared to the other substances and the control group. A possible explanation could be a leakage of the blood–brain barrier which has been described after drug abuse [4, 29] but appears to vary in severity according to the type of substance and remains to be more investigated.

## Limitations

The pilot study is limited by its sample size and the inclusion of heterogeneous study material with a wide age range and differing PMI, which may have influenced the staining intensity and the distribution pattern of the examined cells in the brain. On the other hand, it represents the realistic tissue quality of our daily autopsy material. Moreover, different formaldehyde fixation times of the tissues may have led to different staining intensities erroneously interpreted as different expression patterns of the cells examined. After TMEM119 and CD68KiM1P immunohistochemistry staining, we could observe subtle staining differences in perikaryal and terminal processes. TMEM119 displayed delicate terminal microglial processes, whereas CD68 staining would be the choice for the staining of microglial perikarya. It remains to be clarified whether the lack of clear-cut perikaryal staining in MOR cases and the preservation of finely granular profiles represents a dissociation between protein synthesis or expression of TMEM119 in the perikaryon and long-term location of this transmembrane protein in distal processes. In other words, synthesis is suppressed whereas terminal transmembrane location could be preserved. Alternatively, this dissociation could also represent an artifact caused by extended postmortem or fixation times. Future parallel single-cell mRNA analyses and western blot studies could solve the question of extended postmortem delay on quantitative immunohistochemical investigations.

Finally, although the inclusion of lethal intoxication (MOR, METH) cases was defined as necessarily being monosubstance deaths proven by postmortem toxicological analysis, we cannot exclude co-abuse with other illicit substances during lifetime. In death cases happened under the influence of ethanol (ETH), the history of ethanol abuse has been derived from the clinical (as far as available) and the police records. These data are only an estimation and are based on former anamnesis and statements of witnesses. However, in forensic cases, these sources are routinely used and represent the only possibility to obtain relevant information on this issue in fatalities.

## Conclusion

Our pilot study provides semiquantitative arguments in favor of different glial reactions in MOR- versus ETH- and METH-fatalities.

A significant reduction of TMEM119-positive cell density, especially in the brain cortex, could be observed after MOR intoxications, thus confirming the postulated immunosuppressive effect of morphine. Inherent limitations in postmortem diagnosis of human autopsy tissue warrant a multi-institutional scientific endeavor to provide more human tissue in best possible quality, to reduce confounding artefacts, to disclose the pathogenesis and selective vulnerability of the central nervous system and special cell types associated to drug-induced fatalities, and to assess the complex role of microglia in human health and disease.

## Supplementary Information

Below is the link to the electronic supplementary material.

Supplementary file1 (DOCX 1336 KB)
