# Supplementary material for: Quantitative evidence of suppressed TMEM119 microglial immunohistochemistry in fatal morphine intoxications
Source: Int J Legal Med. 2021 Sep 22;135(6):2315–22. doi: 10.1007/s00414-021-02699-5 (PMC8523458; doi:10.1007/s00414-021-02699-5)
Supplement: Supplementary file 1 — Supplementary file1 (DOCX 1336 KB) [file 414_2021_2699_MOESM1_ESM.docx]

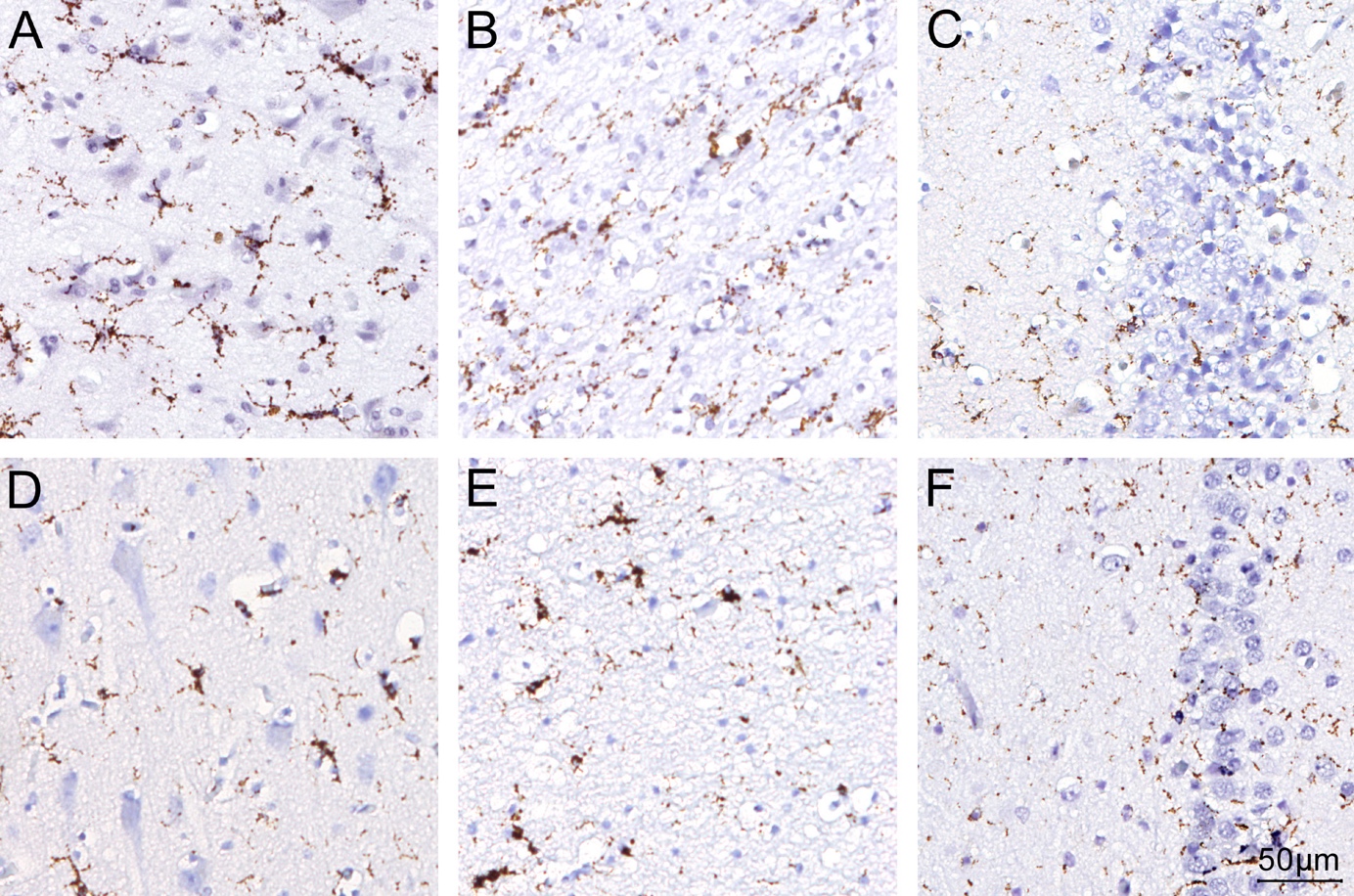


**Supplemental Figure 1:** Representative examples of immunohistochemical staining results using anti-CD68 KiM1P in the cortex (A), the white matter (B) and the hippocampus (C) in a control case without toxicological influence and the cortex (D), the white matter (E) and the hippocampus (F) in fatal morphine intoxication deaths. Magnification: 200x.


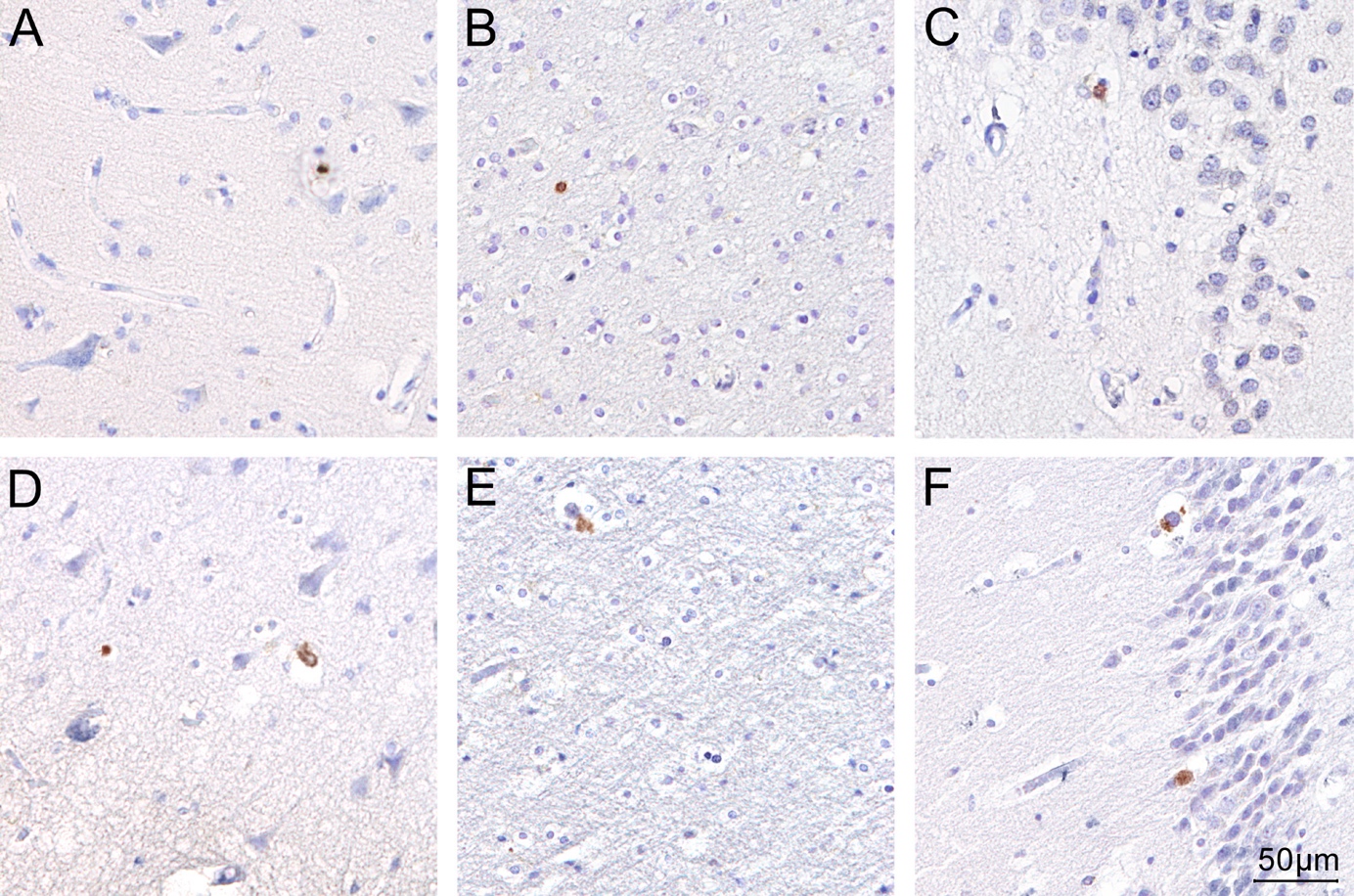


**Supplemental Figure 2:** Representative examples of immunohistochemical staining results using anti-CCR2 in the cortex (A), the white matter (B) and the hippocampus (C) in a control case and the cortex (D), the white matter (E) and the hippocampus (F) in fatal morphine intoxication deaths. Magnification: 200x.
